# Welcome to *Plant‐Environment Interactions*: A new home for international plant and environmental sciences research

**DOI:** 10.1002/pei3.10024

**Published:** 2020-06-10

**Authors:** Wayne Dawson

As Editor‐in‐Chief, it is a pleasure to welcome you to *Plant‐Environment Interactions*, and our very first issue. Our new journal launches at a challenging and tragic time for the world. We all hope the Covid‐19 pandemic ends as quickly as possible, and that all our readers and authors stay safe and well. When the pandemic does end, other global challenges will of course remain. How can we grow drought‐ and pest‐resistant crops to sustainably feed our growing global population and in the face of ongoing climate change? How can we harness molecular tools to produce those crops for the future, and to further our fundamental understanding of how plants interact with their environment? How will wild plant species and communities respond to the effects of climate change, land‐use change, nutrient deposition, and rising CO_2_ levels? What role will interactions with other species play in moderating those responses? Research in the plant and environmental sciences is central to solving these global challenges, and that research will come from across the world.

Our aim at *Plant‐Environment Interactions* is to provide a home for sound, international science to be published in the plant and environmental sciences. *Our scope is global and broad; we will consider work that may not necessarily be novel, but which will advance our understanding of how plants interact with their abiotic and/or biotic environment*. Thus, we will consider work spanning the three categories of plant ecology and evolution, plant physiology, and plant cell and molecular biology. We welcome direct submissions across these three areas, and we are just as happy to consider manuscripts of reviews, new concepts and perspectives, as well as original research articles. We are also keen to consider transferred manuscripts of sound science that may not have been accepted in our partner journals but fit well with our aims and scope at *Plant‐Environment Interactions*. We recognise the valuable efforts made by reviewers of manuscripts and believe that where manuscripts have already been reviewed elsewhere, these reviews should be used as much as possible. At *P‐EI*, we will make use of existing reviews of transferred manuscripts to assist authors in improving their work, and to come to a more rapid decision on manuscripts sent to us. We want to support authors to get their sound science published, rather than simply find reasons not to.

**FIGURE 1:**
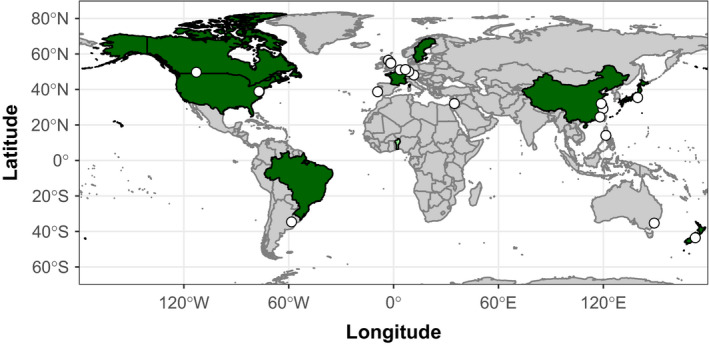
Locations of P‐EI Editorial Board members (circles), countries of corresponding authors for accepted articles (dark green) and countries where research was done if different from author country (pale green)

To reflect our aim of being a home for international research in the plant and environmental sciences, we are building a skilled and experienced Editorial Board team of associate editors from around the world. So far, our Editorial Board members are based in 14 countries on five continents (Figure 1). However, we still have work to do to further diversify the board. We would especially welcome enquiries to join the Board from suitably experienced researchers in the plant and environmental sciences based in Latin America and Africa, and from women (please e‐mail enquiries to PEI@wiley.com). In terms of authorship, we already have accepted articles from authors based in seven countries, with research conducted on six continents (Figure 1). Again, this is a great start for our journal, but we can still do better‐ we will consider manuscripts of sound science that meet our aims and scope from anywhere in the world. Please see our Author Guidelines on our website for information on our aims and scope, and for support and resources to help you prepare your manuscript for submission at *P‐EI*. Waivers and discounts for publication are also available for developing countries (click here for details). Finally, our readership should be international too, and to support this, *P‐EI* is fully open‐access. To support the open access model, Wiley has a number of agreements with funders, institutions and countries to cover article processing charges for authors, meaning many authors do not need to cover the open access fees themselves.

I’m very happy that our first issue meets our aim to publish international research with a broad scope. First, Cordeiro et al. (2020) present a quantitative review of how fine‐root dynamics vary with soil depth and precipitation in a nutrient‐poor Central Amazonian rain forest. Using data from rhizotron observations, Cordeiro et al. show how important deeper roots are in contributing to fine‐root productivity, with implications for our understanding of forest soil carbon stocks and cycling under environmental change. Next, Tisné et al. (2020) explore how the environment regulates the abscission of fruits, focusing on oil palm (*Elaeis guineensis*) in the Benin Republic. Understanding what determines fruit drop is important ecologically for seed dispersal, and economically in terms of production and yield in this economic crop. Moving on to ornamental plants, Song, Zhang, Chen, Zhu, and Wang (2020) demonstrate how growth, physiology and biochemistry of five ornamental plant species respond to lead‐contaminated soils. Song et al.’s findings allow them to recommend which of the ornamental plants are most suitable for potential use in phytoremediation. With a focus on plant physiology, Schützenmeister et al. (2020) show that nitrous oxide (N_2_O) emissions from soils are reduced by photosynthetic activity of two tree species, which may have implications for our understanding of N_2_O flux in terrestrial ecosystems. Finally, Rolando, Gaskin, Horgan, and Richardson (2020) complete the issue with an assessment of how uptake of herbicides by the invasive tree *Pinus contorta* in New Zealand is affected by dose and the application of follow‐up adjuvant treatment. Such information can improve the efficacy of herbicide application aimed at control and eradication of this high‐impact invasive tree, while also reducing the amount of herbicide needed (and hopefully undesired impacts).

I very much enjoyed reading these articles, and I hope you enjoy reading them too. I am excited to see this first issue published and I look forward to seeing the science in our journal grow over the coming months and years.

Stay safe and stay well.
